# Influence of Flavonoid-Rich Fraction of *Monodora tenuifolia* Seed Extract on Blood Biochemical Parameters in Streptozotocin-Induced Diabetes Mellitus in Male Wistar Rats

**DOI:** 10.3390/metabo13020292

**Published:** 2023-02-16

**Authors:** Samuel Nzekwe, Adetoun Morakinyo, Monde Ntwasa, Oluwafemi Oguntibeju, Oluboade Oyedapo, Ademola Ayeleso

**Affiliations:** 1 Department of Biochemistry, Faculty of Science, Adeleke University, Ede 232101, Osun State, Nigeria; 2 Department of Life and Consumer Sciences, University of South Africa, Florida Park, Johannesburg 1709, South Africa; 3 Phytomedicine and Phytochemistry Group, Oxidative Stress Research Centre, Department of Biomedical Sciences, Faculty of Health and Wellness Sciences, Cape Peninsula University of Technology, Bellville 7535, South Africa; 4 Department of Biochemistry, Obafemi Awolowo University, Ife 220282, Osun State, Nigeria; 5 Biochemistry Programme, College of Agriculture, Engineering and Science, Bowen University, Iwo 232102, Osun State, Nigeria

**Keywords:** diabetes mellitus, *M. tenuifolia*, lipid profile, kidney functions, cardiac functions

## Abstract

Diabetes mellitus is a metabolic disorder caused by either the total destruction of the pancreatic beta cells that secrete insulin for the uptake of glucose from the circulation or as a result of the inability of body cells to respond to the presence of insulin in the blood. The present study investigated the effect of a flavonoid-rich fraction of *Monodora tenuifolia* seed extract (FFMTSE) on blood parameters in streptozotocin (STZ)-induced diabetic male Wistar rats. The rats were divided into seven groups (*n* = 6). Group 1: normal control rats, Group 2: rats + FFMTSE (25 mg/kgbwt), Group 3: rats + FFMTSE (50 mg/kgbwt), Group 4: diabetic control rats, Group 5: diabetic rats + FFMTSE (25 mg/kgbwt), Group 6: diabetic rats + FFMTSE (50 mg/kgbwt), and Group 7: diabetic rats + Metformin. The assessment of the lipid profile, kidney functions (urea and creatinine), and cardiac biomarkers (LDH and CK-MB) were carried out in the plasma using established protocols. The results showed a significant increase in the concentrations of triacylglycerol, cholesterol, LDL-cholesterol, VLDL-cholesterol, urea, and creatinine, as well as in cardiac enzyme activities in diabetic rats. However, the administration of the FFMTSE significantly improved the observed biochemical parameters. In addition, an increased concentration of HDL-cholesterol concentration was observed in the diabetic rats upon treatment with FFMTSE. These findings indicate that FFMTSE could be a potent anti-nephropathy and anti-cardiomyopathy agent in diabetic conditions.

## 1. Introduction

Diabetes mellitus is a global disease that results in substantial morbidity, mortality, and long-term complications, including retinopathy, nephropathy, peripheral nerve damage, and cardiovascular diseases [1]. It is characterized by chronic hyperglycemia and impaired metabolism of carbohydrates, fat, and protein associated with an absolute or relative deficiency in insulin secretion or insulin action [2]. Obesity, genetic disposition, sedentary lifestyle, and unhealthy diets are well-known risk factors associated with the development of type 2 diabetes mellitus (T2DM). The International Diabetes Federation (IDF) established that about 415 million adults between the ages of 20 to 79 years were living with diabetes mellitus as of 2015. In addition, diabetes mellitus has proven to be a global public health burden, and it has been projected that the number of diabetic patients will increase to 200 million by 2040 [3]. Diabetes mellitus is characterized by chronic hyperglycemia, which is in synergy with other metabolic aberrations, such as cardiovascular diseases (CVD), obesity, hypertension and fatty liver diseases. These metabolic disorders can cause damage to various organs or tissues, leading to the development of disabling and life-threatening health complications [4]. Medicinal plants contain potent substance(s) that can be used in the treatment of disease(s) or can be used as basic raw materials to produce synthetic drugs [5]. They are rich sources of phytochemicals and bioactive metabolites that can act in synergy as preventive or chemotherapeutics against different diseases or as complementary or alternative therapeutics to modern medicines [6,7]. Recently, there has been an upsurge of interest in the therapeutic potentials of medicinal plants as antioxidants against free radical-induced tissue injury. Besides, well-known and traditionally used natural antioxidants are derived from tea, wine, fruits, vegetables, and spices, while some natural antioxidants are exploited commercially either as antioxidant additives or as nutritional supplements [8]. These bioactive compounds, such as phenols, flavonoids, alkaloids, saponins, tannins, steroids, terpenoids, and stilbenes, in medicinal plants are involved in the management of different diseases [9]. The active ingredients of plants are also extracted and used as raw materials in pharmaceutical industries for the synthesis of drugs [10]. These bioactive compounds possess therapeutic effects, such as blood thinning, antibiotics, anti-malaria, anti-depression, laxative, and anticancer effects [11].

*Monodora* is a genus of plants in the family ‘Annonacaea.’ The species of *Monodora* include *Monodora myristica* and *Monodora tenuifolia* Benth, which are widely used as spices [12]. It is a plant endowed with rich ethnobotanical history, and its medicinal properties have been reported [13,14]. In traditional medicine practice, it is widely used against toothache, dysentery, diarrhea, dermatitis, headache, and parasitic worms [15,16]. The seeds are aromatic and used as an ingredient in herbal medicines in Southern Nigeria. In the food industry, the seeds are used as spices in condiments and flavors; also, when roasted, the ground seeds are rubbed on the skin for skin diseases [17,18]. The present study investigated the effects of a flavonoid-rich fraction of *Monodora tenuifolia* seeds extract (FFMTSE) on some biochemical parameters in the blood of streptozotocin-induced diabetic male Wistar rats.

## 2. Materials and Methods

### 2.1. Collection of Plant Materials

The fruits of *Monodora tenuifolia* were collected from the Botanical Garden, Obafemi Awolowo University (OAU), Ile-Ife, Nigeria. It was identified by a botanist in the Department of Botany, Faculty of Science, Obafemi Awolowo University (OAU), Ile-Ife, and deposited at the IFE Herbarium with an identification number (IFE17979). The fruits were cut open to remove the hard-coated seeds and dried. Next, the dried seeds were deshelled to remove the seed coat, followed by the grinding of the seeds to fine powder with an electronic blender according to the method of Akinwunmi and Oyedapo [19]. The powdered seed was soaked in 80% (*v/v*) ethanol in the ratio of 1:5 (*w/v*) for 48 h and filtered using a clean cellophane material and white cotton wool, followed by concentration to a thick slurry in a rotary evaporator.

### 2.2. Reagents and Chemicals

All reagents used in the study were of analytical grade and obtained from various sources. Ethanol, n-hexane, and ethylacetate were from Fisher Scientific U.K, Merck KGaA, Germany, and Guandang Guanghan Chemical, China, respectively. D-fructose was from Mumbai, India, HCl was from Mumbai, India, and disodium hydrogen phosphate and monosodium dihydrogen phosphate were from Guandong, China. Other reagents, such as sodium citrate, trichloroacetic acid, hydrogen peroxide, aluminum chloride, and sodium nitrite, were all bought from BDH laboratories in Poole, UK. Diagnostic kits for plasma urea, plasma creatinine, and lipid profile were purchased from Randox Laboratories Ltd., UK. Diagnostic kits for CK-MB and LDH were purchased from Biorex diagnostics UK.

### 2.3. Preparation of Flavonoids-Rich Fraction of the Extract

Hydro-ethanolic extract of *Monodora tenuifolia* seeds was partitioned using a solvent-solvent extraction method according to Akinwunmi and Oyedapo [20] as reported by Chukwuma and Chiamaka [21] with slight modification.

The extract was dissolved in hot distilled water in a ratio of 1:5 (*w/v*). Next, the solution was hydrolyzed with 1% H_2_SO_4_ (1:4 *v/v*) by refluxing using a condensation assembly mounted on a magnetic stirrer for 5 min and filtered after cooling on an ice pack to obtain the filtrate. The filtrate was mixed thoroughly with ethylacetate (1:4 *v/v*) successively and poured into a separating funnel, and it was then allowed to settle. The ethylacetate fraction of the extract was then concentrated in a rotary evaporator and used as the flavonoid-rich fraction, according to the work of Gupta et al. [22] and Bala et al. [23].

### 2.4. Determination of Total Flavonoid Content

The total flavonoid concentration in the ethylacetate fraction of *M. tenuifolia* seed extract was determined using the method of Kostic et al. [24] with slight modifications, with rutin as the standard. Rutin (1 mg/mL) was prepared in methanol-distilled water (1:1 *v/v*) to form the stock solution, then further diluted into 6 serial dilutions of 0–1000 µL and made up to 1 mL with distilled water. The dilutions (200 µL) and sample (5 mg/mL) were pipetted separately and added to 300 µL of freshly prepared 5% NaNO_3_, followed by the addition of 300 µL of 10% AlCl_3_ and then 1 mL of 4% NaOH. After incubation at 25 °C for 10 min, the absorbance of the reaction mixture was read at 500 nm. The standard calibration graph was plotted, and the concentrations of flavonoids were determined and are expressed as mgRE/g extract.

where, RE = Rutin Equivalent.

### 2.5. Collection of Experimental Animals

Forty-two (42) male Wistar rats weighing 150–200 g were purchased from the Department of Anatomy, LAUTECH, Ogbomoso, Nigeria. The animals were acclimatized for 2 weeks in the animal House, Department of Biochemistry, Adeleke University and maintained under 12 h light/dark cycle in normal conditions of temperature and humidity. They were fed with standard pellets and water ad-libitum according to the method described by Hamid et al. [25].

### 2.6. Induction of Diabetes Using Streptozotocin (STZ)

Twenty-four (24) male Wistar rats were fed with 10% fructose in drinking water for 2 weeks before the intraperitoneal administration of a single dose of streptozotocin (STZ) (40 mg/kg bwt) injection [26]. Diabetes was confirmed using an Acu-check glucometer 72 h after induction, and the rats with ≥250 mg/dL glucose level were considered diabetic. The diabetes-induced groups were allowed to stabilize for 21 days with routine checks for glucose levels at 7-day intervals using an Acu-check glucometer.

### 2.7. Grouping and Treatment of Experimental Animals

The rats were divided into seven groups of six (6) rats in each group.

Group 1: Normal control rats

Group 2: Normal rats + 25 mg/kg FFMTSE

Group 3: Normal rats + 50 mg/kg FFMTSE

Group 4: Diabetic control rats

Group 5: Diabetic rats + 25 mg/kg FFMTSE

Group 6: Diabetic rats + 50 mg/kg FFMTSE

Group 7: Diabetic rats + 500 mg/kg Metformin

After 21 days of maintaining diabetes in the rats, plant extracts (25 mg/kg FFMTSE and 50 mg/kg FFMTSE) and standard drugs (metformin, 500 mg/kg) were administered in a single dose daily. Group 1 served as the control group taking water and feed only. Groups 2 and 3 were normal rats that received 25 mg/kg and 50 mg/kg FFMTSE, respectively. Group 4 served as the untreated diabetic (diabetic control) group, while groups 5 and 6 were diabetic groups that were administered 25 mg/kg and 50 mg/kg FFMTSE, respectively. Group 7 was the diabetic group treated with 500 mg/kg metformin. The treatments were administered in single doses of the extract and metformin daily in 1ml for 14 days.

### 2.8. Sacrificing of Experimental Animals and Blood Collection

At the end of the experimental period, the rats were sacrificed after overnight fasting using diethylether anesthesia according to the method of Akinwunmi and Oyedapo [19]. The blood samples were collected through venous punctures using different anticoagulant (lithium heparin)-coated vials and kept on ice. The blood samples were then centrifuged at 4000 rpm in centrifuge 800D, and the plasma was separated and frozen at 20 °C for subsequent biochemical analysis.

### 2.9. Determination of Lipid Profile

#### 2.9.1. Estimation of Total Cholesterol Concentration

Cholesterol concentration was determined according to the procedure described in the Randox manufacturer’s instructional manual.

#### 2.9.2. Estimation of Triglycerides Concentration

Triglyceride concentration was determined using a kit according to the procedure described in the Randox manufacturer’s instructional manual.

#### 2.9.3. Determination of High-Density Lipoprotein Cholesterol (HDL-c) Concentration

The concentration of HDL-cholesterol (HDL-c) concentration was determined according to the procedure described in the Randox manufacturer’s instructional manual.

#### 2.9.4. Estimation of Low-Density Lipoprotein Cholesterol (LDL-c) Concentration

The concentration of plasma low-density lipoprotein cholesterol (LDL-c) was evaluated using Friedewald’s formula according to the expression below [27].

$$\mathrm{LDL-}c\;(\mathrm{mg}/\mathrm{dL})=\mathrm{Total}\;\mathrm{Cholesterol}-(\frac{\mathrm{Triglycerides}}{5}+\;\mathrm{HDL}-\mathrm{cholesterol})$$

#### 2.9.5. Estimation of Very Low-Density Lipoprotein Cholesterol (VLDL-c) Concentration

The plasma concentration of VLDL-cholesterol (VLDL-c) was calculated according to Friedewald’s equation, expressed below [27]:

$$\mathrm{VLDL-}c\;(\mathrm{mg}/\mathrm{dL})=\frac{\mathrm{Triglycerides}\;}{5}$$

### 2.10. Determination of the Concentrations of Renal Biomarkers

#### 2.10.1. Estimation of Plasma Urea Nitrogen Concentration

Blood urea nitrogen (BUN) concentration was estimated by the method of Fawcett and Scott [28] using the commercially available kit. The absorbance of test and standard samples was measured spectrophotometrically against blank at 578 nm and expressed as mg/dL.

$$\mathrm{Urea}\;\mathrm{concentration}\;(\mathrm{mg}/\mathrm{dL})=\frac{\mathrm{Abs}.\;\mathrm{of}\;\mathrm{Sample}\;\times \;\mathrm{Conc}.\;\mathrm{of}\;\mathrm{Standard}\;\left(\mathrm{mg}/\mathrm{dL}\right)\;}{\mathrm{Abs}.\;\mathrm{of}\;\mathrm{Standard}}$$

#### 2.10.2. Estimation of Plasma Creatinine Concentration

The creatinine concentration was estimated by the alkaline picrate method, as described by Bonsnes and Taussky [29], using a commercially available diagnostic kit. The absorbance was measured at 510 nm against distilled water and expressed as mg/dL.

$$\mathrm{Creatinine}\;\mathrm{concentration}\;(\mathrm{mg}/\mathrm{dL})=\frac{\mathrm{Abs}.\;\mathrm{of}\;\mathrm{Sample}\;\times \;\mathrm{Conc}.\;\mathrm{of}\;\mathrm{Standard}\;\left(\mathrm{mg}/\mathrm{dL}\right)\;}{\mathrm{Abs}.\;\mathrm{of}\;\mathrm{Standard}}$$

### 2.11. Determination of Cardiac Biomarkers in Plasma

#### 2.11.1. Assay of Creatinine Kinase-Myocardial Band [CK-MB] Activity

The assay of CK-MB activity was carried out according to the method of Jansson and Sylvén [30]. Briefly, 1 mL of the working reagent was pipetted into test tubes containing 40 µL of plasma, followed by incubation at 37 °C for 3 min. The mixture was measured at 475 nm in intervals of 1 min for 5 min.

$$\mathrm{CK-}\mathrm{MB}\;\mathrm{activity}\;(\mathrm{mg}/\mathrm{dL})=\frac{\mathrm{Abs}.\;\mathrm{of}\;\mathrm{Sample}\;\times \;\mathrm{Conc}.\;\mathrm{of}\;\mathrm{Standard}\;\left(\mathrm{mg}/\mathrm{dL}\right)\;}{\mathrm{Abs}.\;\mathrm{of}\;\mathrm{Standard}}$$

#### 2.11.2. Assay of Lactate Dehydrogenase (LDH) Activity

The assay for the LDH activity was carried out according to the method of Bernstein [31] using a commercially available kit. The plasma (40 µL) was added to l mL of the working reagent and incubated at 37 °C for 3 min. The absorbance was measured at 340 nm within the interval of 30 s for 150 s. The activity was calculated as shown below, and the result was expressed in mmol/L.

(1) $$\mathrm{LDH}\;\mathrm{activity}\;(\mathrm{mmol}/L)=\frac{\mathrm{Abs}.\;\mathrm{of}\;\mathrm{Sample}\;\times \;\mathrm{Conc}.\;\mathrm{of}\;\mathrm{Standard}\;\left(\mathrm{mmol}/L\right)}{\mathrm{Abs}.\;\mathrm{of}\;\mathrm{StandAbs}.\;\mathrm{of}\;\mathrm{Standardard}}$$

### 2.12. Statistical Analysis

The data were expressed as mean ± standard deviation (SD) of triplicates. Statistical significance was determined by a 1-way analysis of variance (ANOVA), followed by Duncan’s multiple comparisons between control and treated rats in all the groups.

## 3. Results

### 3.1. Total Flavonoid Concentration in FFMTSE

Table 1 shows the concentration of flavonoids present in 1 mg of the FFMTSE extracted with ethylacetate. One (1 mg) of the extract contained 0.078 mg of flavonoid expressed in standard rutin equivalent with a standard deviation of 0.001.

### 3.2. Effect of FFMTSE on Lipid Profile

**Figure 1:**
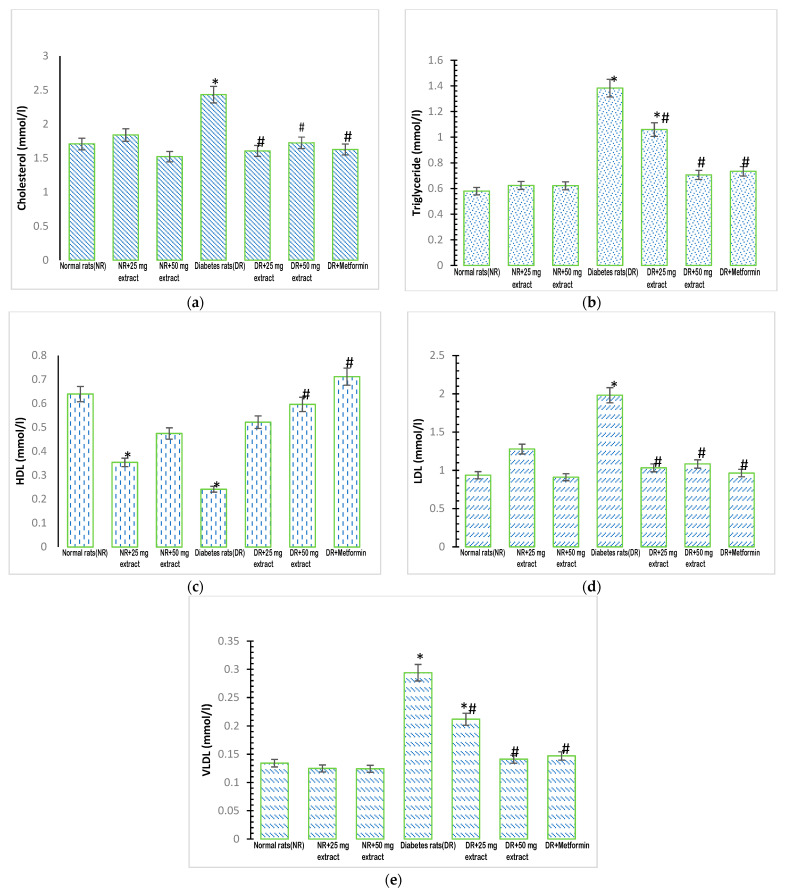
Lipid profile of rats treated with FFMTSE. (**a**) Concentration of cholesterol, (**b**) triglycerides, (**c**) HDL-Cholesterol, (**d**) LDL cholesterol, and (**e**) VLDL cholesterol in rats treated with FFMTSE. All results are expressed as mean ± SD. (*) indicates a significant difference compared to the normal control group at *p* < 0.05, and (#) indicates a significant difference compared to the diabetic control group at *p* < 0.05. FFMTSE- Flavonoid-rich fraction of *Monodora tenuifolia* seed extract.

The concentration of plasma cholesterol was significantly higher (*p* < 0.05) in the diabetic control group (Figure 1a) than in the normal control group. However, when the diabetic groups were treated with the plant extracts (25 mg/kg FFMTSE and 50 mg/kg FFMTSE) and metformin, there was a significant reduction in cholesterol concentrations compared with that in the diabetic control group.

The concentration of plasma triglycerides in the diabetic control group increased significantly (*p* < 0.05) compared with that in the normal control group (Figure 1b). Treatment of the diabetic rats with the extracts (25 mg/kg FFMTSE and 50 mg/kg FFMTSE) and metformin resulted in a significant decrease in plasma triglyceride concentrations compared with that in the diabetic control group.

There was a significant reduction (*p* < 0.05) in the concentration of plasma HDL-cholesterol in the diabetic control group (Figure 1c) compared with that in the normal control group. However, when the diabetic groups were treated with the plant extract (50 mg/kg FFMTSE) and metformin, there was a significant increase in HDL-cholesterol concentrations compared with that in the diabetic control group.

The concentration of plasma LDL-cholesterol in the diabetic control group increased significantly (*p* < 0.05) compared with that in the normal control group (Figure 1d). When the diabetic groups were treated with the plant extracts (25 mg/kg FFMTSE and 50 mg/kg FFMTSE) and metformin, the results showed a significant decrease in LDL-cholesterol concentrations compared with that in the diabetic control group.

The concentration of plasma VLDL was significantly increased (*p* < 0.05) in the diabetic control group (Figure 1e) when compared with that in the normal control group. Treatment with the extracts (25 mg/kg FFMTSE and 50 mg/kg FFMTSE) and metformin led to a significant reduction in VLDL concentrations in the treated diabetic groups compared with the diabetic control group. However, VLDL concentrations in the diabetic rats treated with 25 mg/kg FFMTSE were significantly higher than that in the normal rats.

### 3.3. Effect of FFMTSE on Kidney Biomarkers

**Figure 2:**
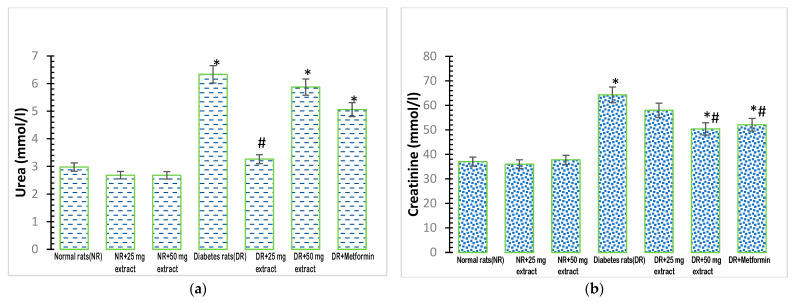
Kidney biomarkers in rats treated with FFMTSE. (**a**) Concentration of urea and (**b**) creatinine in rats treated with FFMTSE. All results are expressed as mean ± SD, (*) indicates a significant difference compared to the normal control group at *p* < 0.05, and (#) indicates a significant difference compared to the diabetic control group at *p* < 0.05. FFMTSE: flavonoid-rich fraction of *Monodora tenuifolia* seed extract.

Plasma urea concentrations showed a significant increase (*p* < 0.05) in the diabetic control group (Figure 2a), compared with that in the normal control group. The administration of 25 mg/kg FFMTSE to the diabetic rats caused a significant reduction in urea concentrations compared with that observed in the diabetic control group. However, the urea concentrations in diabetic rats treated with 50 mg/kg FFMTSE and metformin were significantly higher than that in the normal rats.

Plasma creatinine concentration was significantly increased (*p* < 0.05) in the diabetic control group, compared with that in the normal control group (Figure 2b). Moreover, 50 mg/kg FFMTSE and metformin significantly decreased creatinine concentrations in diabetic rats, while those treated with 25 mg/kg FFMTSE showed no significant reduction in creatinine concentration. In addition, creatinine concentrations in diabetic rats treated with 50 mg/kg FFMTSE and metformin were significantly higher than that in the normal rats.

### 3.4. Effect of FFMTSE on Cardiac Biomarkers

**Figure 3:**
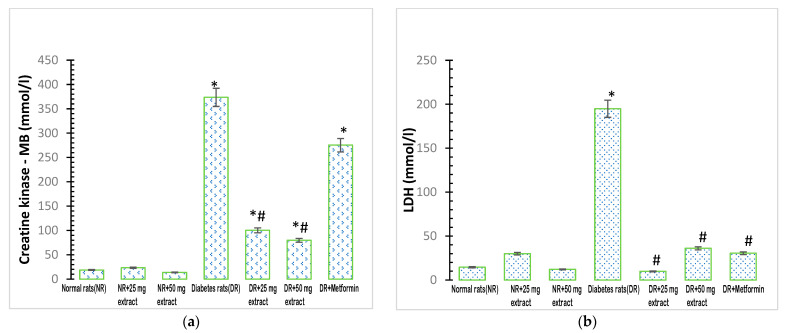
Cardiac biomarkers in rats treated with FFMTSE. (**a**) Activity of creatine kinase-MB (CK-MB) and (**b**) Lactate dehydrogenase (LDH) in rats treated with FFMTSE. All results are expressed as mean ± SD, (*) indicates a significant difference compared to the normal control group at *p* < 0.05, and (#) indicates a significant difference compared to the diabetic control group at *p* < 0.05. FFMTSE: flavonoid-rich fraction of *Monodora tenuifolia* seed extract.

The activity of plasma creatine kinase MB was significantly increased (*p* < 0.05) in the diabetic control group when compared with that in the normal control group (Figure 3a). In addition, the administration of *M. tenuifolia* extracts (25 mg/kg FFMTSE and 50 mg/kg FFMTSE) to the diabetic rats significantly decreased creatine kinase activity, compared with that in the diabetic control group, while creatine kinase activity in the diabetic rats treated with 25 mg/kg FFMTSE and 50 mg/kg FFMTSE as well as metformin were significantly higher than that in the normal rats.

There was a significant increase (*p* < 0.05) in the activity of plasma LDH in the diabetic control group compared with that in the normal control group (Figure 3b). However, the administration of the extracts (25 mg/kg FFMTSE and 50 mg/kg FFMTSE) to the diabetic rats significantly reduced the activity of LDH compared with that in the diabetic control group.

## 4. Discussion

In this study, flavonoid was found in the ethylacetate fraction of *Monodora tenuifolia* seeds, which supports the findings of Akinwunmi and Oyedapo [20] and Ekanyanwu and Njoku [16], who have previously shown that *Monodora tenuifolia* and its family species are rich in flavonoids. Diabetic patients have impaired lipid metabolism (dyslipidemia), accompanied by the risk of cardiovascular arteriosclerosis [32]. Our findings also revealed accumulated levels of lipids in diabetic rats, suggesting that impaired lipid metabolism is associated with diabetes. Type 2 diabetes occurs when body cells or organs are insensitive to the presence of insulin in circulation, and pancreatic β-cells fail to produce enough insulin to compensate for the ongoing insulin resistance. It is closely correlated with dyslipidemia, characterized by increased levels of LDL and triglycerides as well as low levels of HDL [33,34]. The clinical analysis of autopsy specimens carried out by Regan and coworkers on diabetic persons demonstrated increased deposition of cholesterol and triglycerides compared with those observed in persons without the disease [35]. In patients with type 2 diabetes, hyperinsulinemia, insulin resistance, and β-cell failure are related to diabetes dyslipidemia due to elevated plasma levels of fasting triglyceride-rich lipoproteins, small-dense LDL-particles, and low levels of high-density lipoprotein (HDL) cholesterol [36]. The present study showed increased concentrations of LDL-c, triglyceride, VLDL, and cholesterol with reduced concentrations of HDL-c in the plasma of diabetic rats. Treatment with the plant extracts at both test concentrations (25 mg/kg FFMTSE and 50 mg/kg FFMTSE) significantly reduced the concentrations of LDL-c, triglycerides, VLDL, and cholesterol. This indicates that *Monodora tenufolia* seeds have the potential to control lipid metabolism in diabetic conditions. When the diabetic groups were treated with 50 mg/kg FFMTSE and metformin, there was a significant increase in HDL-cholesterol concentrations compared with that in the diabetic control group; however, 25 mg/kg FFMTSE did not show any significant increase in HDL-cholesterol. The effects of the drug on the bodies of animals can be influenced by different factors, including the route of administration, drug concentration, and internal body homeostatic changes. The lower dosage of the plant extract at 25 mg/kg FFMTSE could have caused poor synthesis or metabolism of HDL in the plasma of the rats.

Diabetic nephropathy is a leading cause of chronic and end-stage renal disease, and it is relatively associated with elevated cardiovascular disease risk and mortality. Prolonged hyperglycemia and increased lipid mobilization and accumulation cause glucotoxicity and lipotoxicity, which are associated with chronic renal disease in diabetes. This present study showed significantly increased concentrations of creatinine and urea in diabetic rats, indicating serious diabetes-induced kidney damage. Increased serum levels of lipids in the kidneys of patients with diabetes and experimental animals have been reported [37,38]. The administration of plant extracts (25 mg/kg FFMTSE and 50 mg/kg FFMTSE) significantly reduced urea concentration in diabetic rats. However, only 50 mg/kg FFMTSE was able to reduce creatinine concentration in the diabetic rats, as 25 mg/kg FFMTSE showed no significant decrease in the creatinine concentration. High creatinine level in plasma is an indication of kidney malfunction; the plant extract was expected to lower creatinine levels in diabetic rats and enhance the glomerular filtration of the kidney. The non-significant effect exerted by 25 mg/kg FFMTSE could be attributed to the inability of the extract at this dose to confer any effect on glomerular filtration in the kidney. Nevertheless, at 50 mg/kg, FFMTSE significantly improved the creatinine level and enhanced glomerular filtration in the kidney.

Cardiac lipid accumulation commonly occurs in type 2 diabetes and has been suggested to play a direct causal role in the development of cardiomyopathy and heart failure in a process known as cardiac lipotoxicity [39]. Fatty acid mobilization and a significant increase in cholesterol availability in plasma are some of the characteristics of diabetes mellitus [40]. In this study, there was a significant increase in the activities of cardiac enzymes (LDH and CK-MB) in diabetic rats, which indicated structural damage, functional alteration in the heart, and the excessive release of these enzymes into the bloodstream. The present work supports the finding of Sharma et al. [41], who showed that intra-myocardial lipid overload in heart disease is greater in people with diabetes than in those without diabetes. Cardiac dysfunction induced by the excess accumulation of lipids, termed lipotoxic cardiomyopathy or fatty heart, demonstrates the importance of lipids in the development of diabetic cardiomyopathy and heart failure [42]. Cardiac dysfunction observed in individuals with diabetes is a net result of both lipid-driven cardiac dysfunction due to the effects of lipids on the cardiovascular as well as a direct pathological effect of lipids on the myocardium-promoting cardiomyopathy [43]. FFMTSE (25 and 50 mg/kg) significantly reduced the activities of cardiac enzymes (LDH and CK-MB) in diabetic rats.

## 5. Conclusions

Medicinal plants have been a promising source of phytochemicals that possess medicinal potential in the treatment of many diseases. This study demonstrated the capacity of FFMTSE to influence lipid metabolism through the reduction of blood concentrations of LDL-cholesterol, triglycerides, and VLDL-cholesterol and the improvement of HDL-cholesterol levels in diabetic rats. In addition, kidney parameters (creatinine and urea concentrations) and activities of cardiac enzymes improved in diabetic rats upon treatment with FFMTSE. Overall, the study showed that the flavonoid-rich fraction of *Monodora tenuifolia* seed could help to ameliorate complications arising from kidney and cardiac damage in diabetic conditions. Further investigations may be required to determine the mechanisms of the actions of the flavonoids in *Monodora tenuifolia* against kidney and cardiac dysfunctions in diabetes mellitus.

## Figures and Tables

**Table 1:** Concentration of total flavonoid in FFMTSE.

Plant Sample	Concentration (mgRE/mg FFMTSE)
Flavonoid-rich fraction of *M. tenuifolia*	0.078 ± 0.001

## Data Availability

Samples of the extract are available from the authors.
